# Supplementary material for: A cationic motif upstream Engrailed2 homeodomain controls cell internalization through selective interaction with heparan sulfates
Source: Nat Commun. 2023 Apr 10;14:1998. doi: 10.1038/s41467-023-37757-6 (PMC10083169; doi:10.1038/s41467-023-37757-6)
Supplement: Supplementary file 1 — Supplementary Information [file 41467_2023_37757_MOESM1_ESM.pdf]

Supplementary Information to

A cationic motif upstream Engrailed2 homeodomain controls cell internalization through selective interaction with heparan sulfates

Sébastien Cardon[1#], Yadira P. Hervis[1#], Gérard Bolbach[1,2], Chrystel Lopin-Bon[3], Jean-Claude Jacquinet[3], Françoise Illien[1], Astrid Walrant[1], Delphine Ravault[1], Bingwei He[1], Laura Molina[1], Fabienne Burlina[1], Olivier Lequin[1], Alain Joliot[4], Ludovic Carlier[1,*], Sandrine Sagan[1,*]

Affiliations

[1]Sorbonne Université, École normale supérieure, PSL University, CNRS, Laboratoire des Biomolécules (LBM), 75005 Paris, France.

[2]Sorbonne Université, Mass Spectrometry Sciences Sorbonne University, MS3U platform, 75005 Paris, France.

[3]Univ. Orléans, CNRS, ICOA, 45067 Orléans, France.

[4]INSERM U932, Institut Curie Centre de Recherche, PSL Research University, Paris, France

Keywords
Homeoproteins, Engrailed-2, protein-GAG interaction, membrane translocation,

***Corresponding Authors**
Ludovic.Carlier@sorbonne-universite.fr
Sandrine.Sagan@sorbonne-universite.fr

[#] These authors contributed equally to the work

Table of contents

Screening of ExtHD binding to HS oligosaccharides using a microarray analysis 3

Dynamic light scattering (DLS) analysis of heparin/ExtHD complexes.................................6

Interaction of En2 proteins with GAGs by Isothermal Titration Calorimetry (ITC)........... 7

Investigation of the GAG-binding properties of En2 proteins by NMR.............................. 12

Model for the role of En2-GAG interaction in retinal axon guidance..................................16

Quantification of protein internalization by Mass Spectrometry...17

Confocal imaging of En2 full-length protein internalization 24

References.. 25

Screening of ExtHD binding to HS oligosaccharides using a microarray analysis

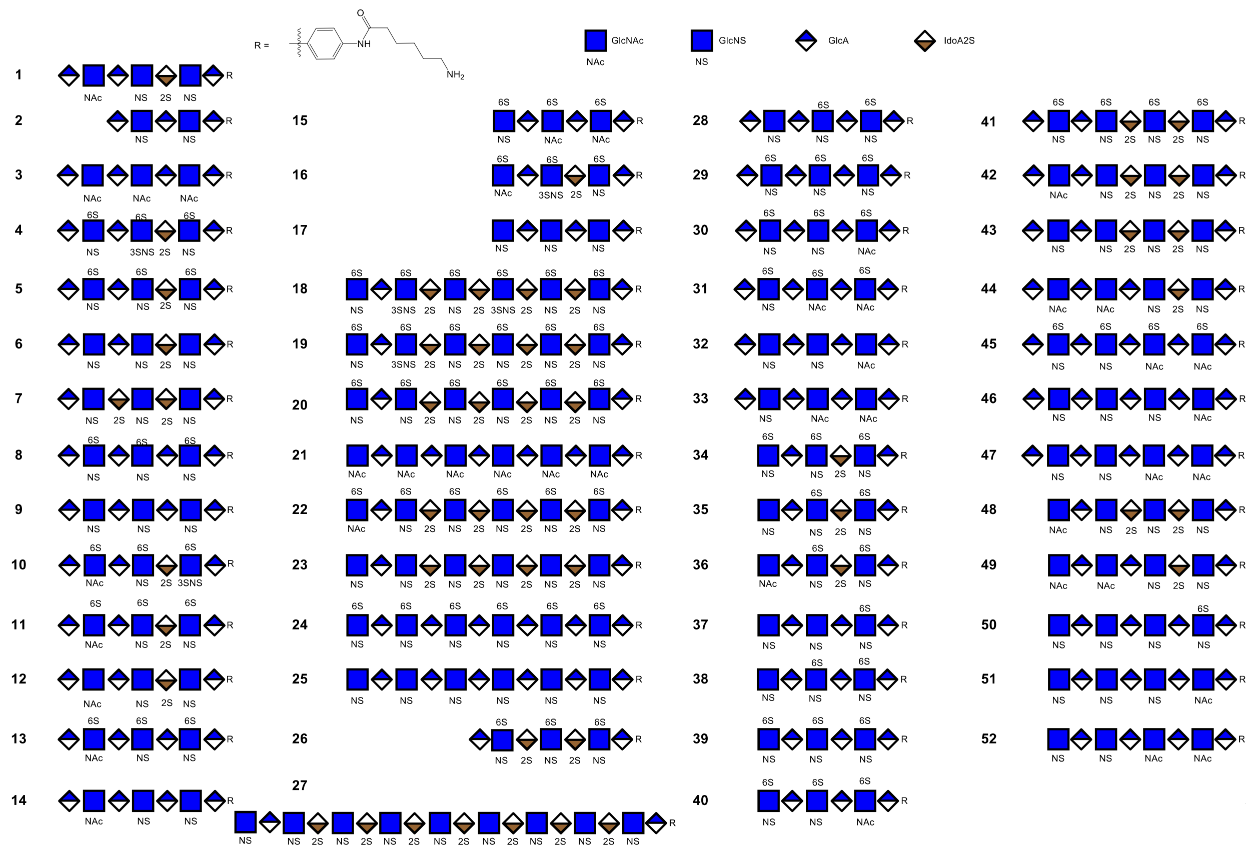

Supplementary Figure 1. Structure of heparan sulfate oligosaccharides used in the microarray analysis to probe the binding to ExtHD. The 52 compounds exhibit different sulfation patterns usually found in nature, with a size range from 5 (5-mer) to 18 (18-mer) monosaccharide units. (https://www.glycantherapeutics.com/services/microarray-analysis).

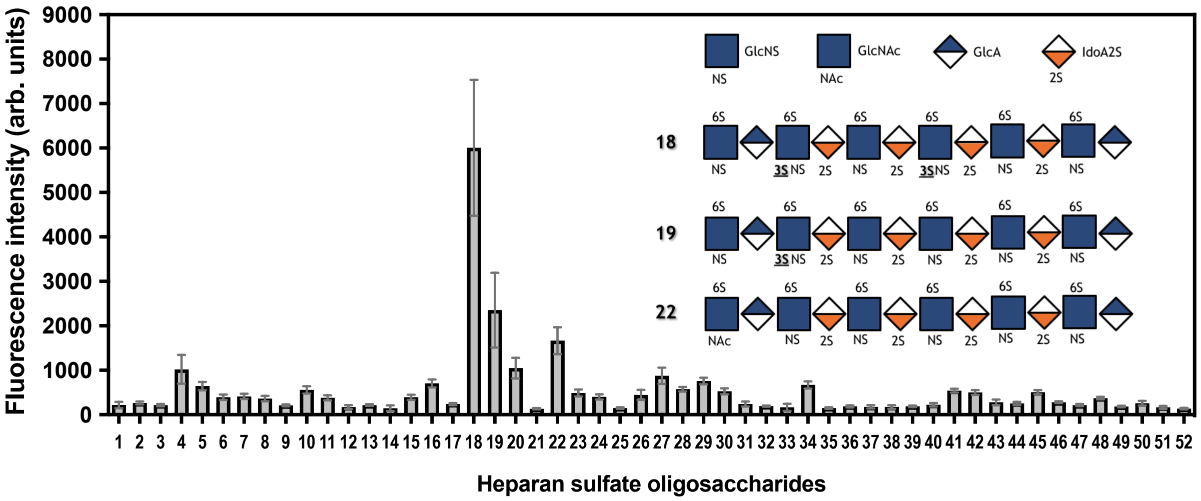

Supplementary Figure 2. Microarray screening data for ExtHD binding to 52 different heparan sulfate compounds. Biotin-labeled ExtHD (100 nM) was incubated on the microarray with different HS structures and its binding revealed by fluorescent streptavidin. The intensity data is the mean value ± SD of 36 individual spots of one microarray screening experiment.

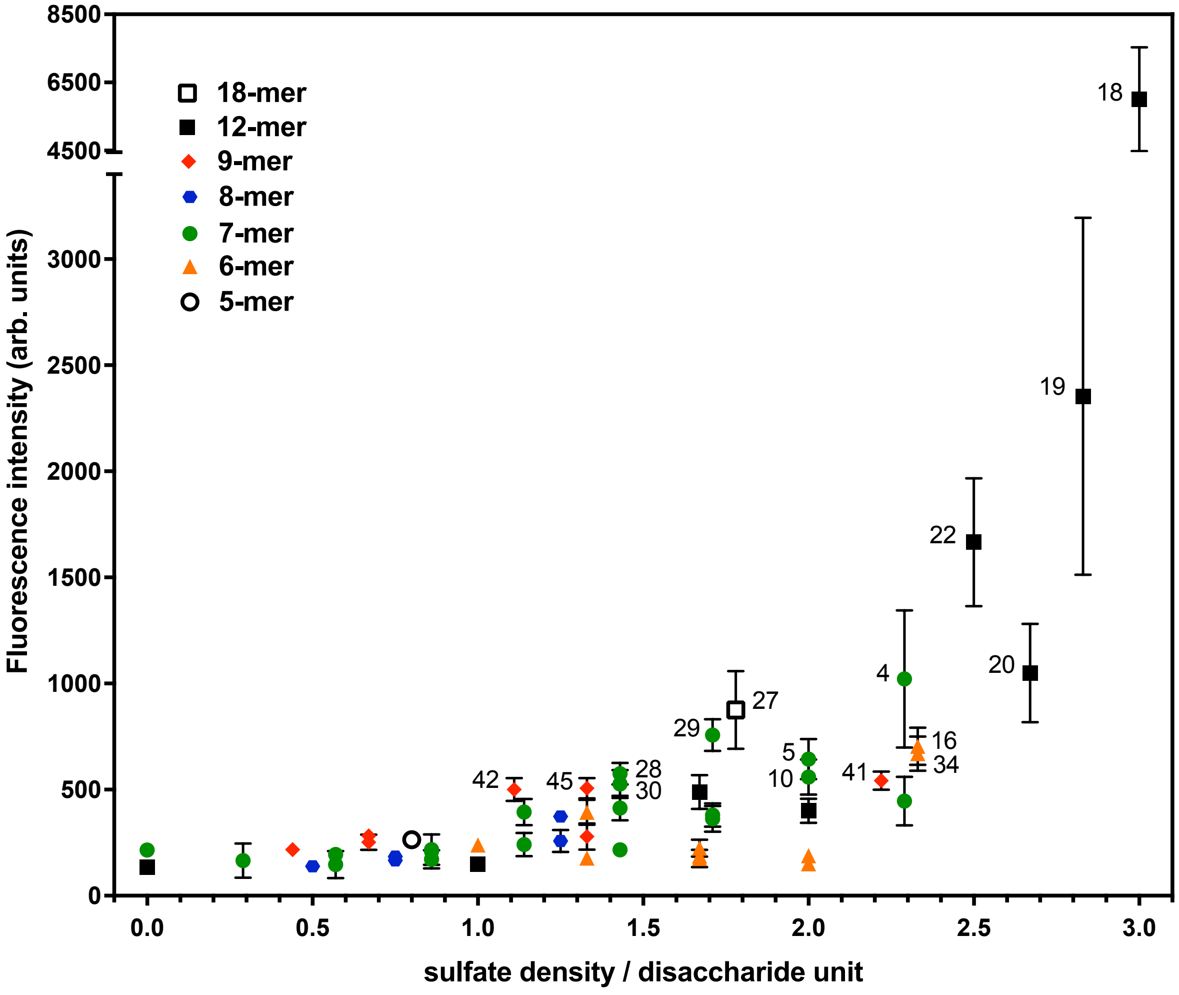

Sulfate density of the HS compounds

Supplementary Figure 3. Scatter plot of the measured fluorescence intensity vs. sulfate density (number of sulfates per disaccharide unit) of the 52 HS oligosaccharides used in the microarray analysis to probe ExtHD binding. Compound numbers are indicated for HS oligosaccharides showing fluorescence intensity higher than 500 arbitrary units (arb. units). The fluorescence intensity is the mean value ± SD of 36 individual spots of one microarray screening experiment.

Dynamic light scattering (DLS) analysis of heparin/ExtHD complexes

	Sample	GAG/ExHD molar ratio	Size (d. nm)	PdI
(black)	ExtHD	-	2.9 ± 0.1	0.51
(red)	ExtHD + 10 µg/mL heparin	0.12	871 ± 75	0.55
(blue)	ExtHD + 100 µg/mL heparin	1.20	7.4 ± 0.6	0.63
(light green)	ExtHD + 10 µg/mL HI dp20	0.25	3.6 ± 1.2	0.55
(orange)	10 µg/mL heparin	-	0.7 ± 0.1	0.88
(pink)	100 µg/mL heparin	-	0.9 ± 0.1	0.64
(dark green)	10 µg/mL HI dp20	-	0.7± 0.1	0.59

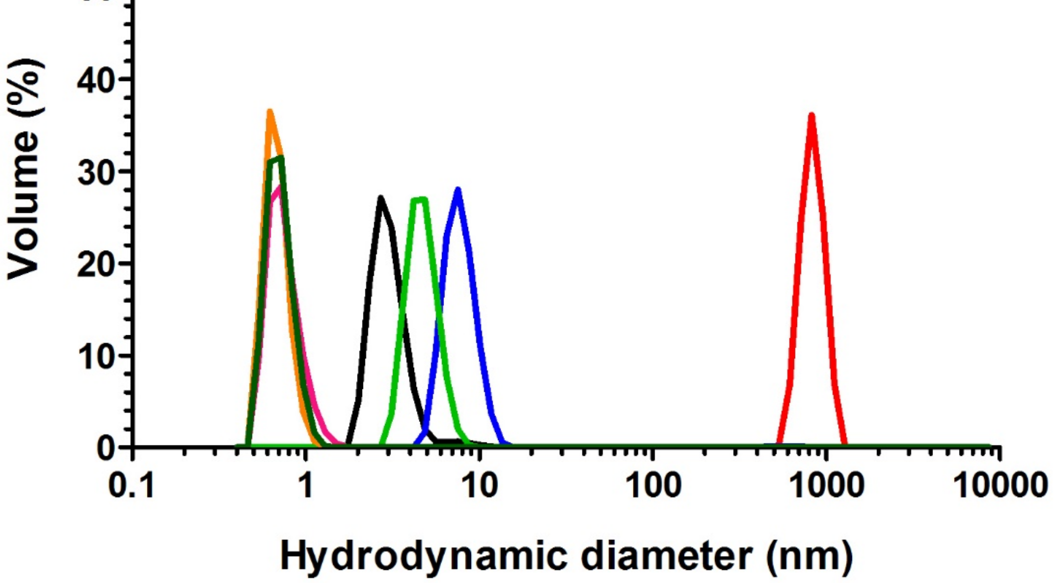

Supplementary Figure 4. Size distribution of ExtHD mixed with full-length heparin and HI dp20. Each sample mix was incubated 1 hr at 37°C before recording the mass distribution using a Zetasizer Nano ZS apparatus (Malvern Instruments, Malvern, UK) : ExtHD alone (black), ExtHD + 10 µg/mL heparin (red), ExtHD + 100 µg/mL heparin (blue), ExtHD + 10 µg/mL HI dp20 (light green), 10 µg/mL heparin (orange), 100 µg/mL heparin (pink), 10 µg/mL HI dp20 (dark green). The table shows the GAG/ExtHD molar ratio for each sample, the size in nanometer, and the polydispersity index (PdI) of particle populations. The size is shown as a mean ± SD (two repeated measurements of the same sample).

Interaction of En2 proteins with GAGs by Isothermal Titration Calorimetry (ITC)

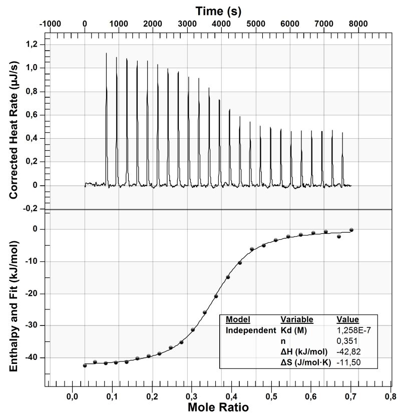

ExtHD

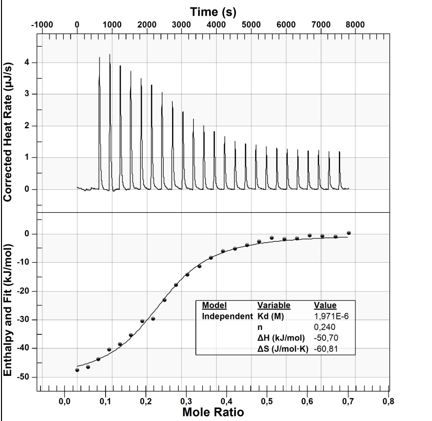

HD

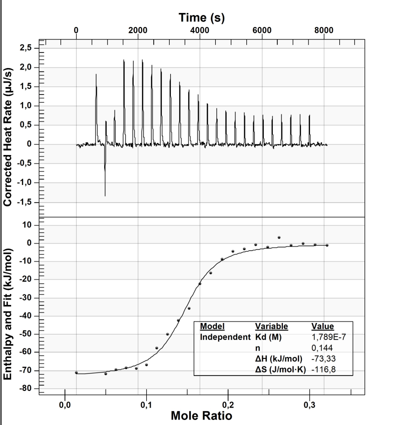

N-ter octadecapeptide

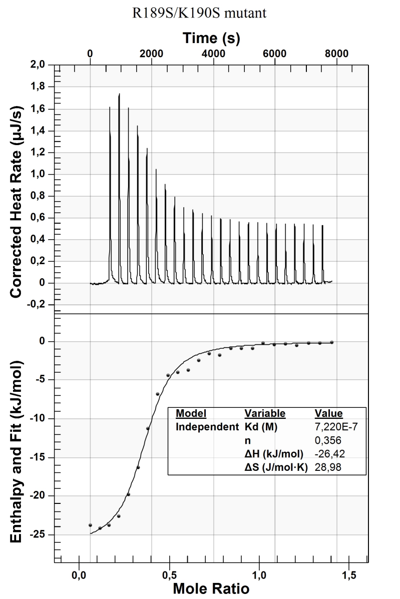

[R189S/K190S]ExtHD

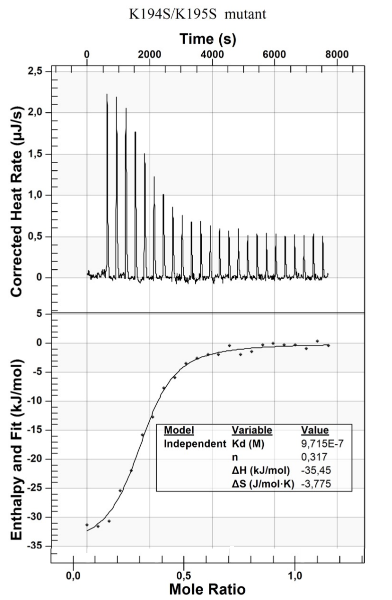

[K194S/K195S]ExtHD

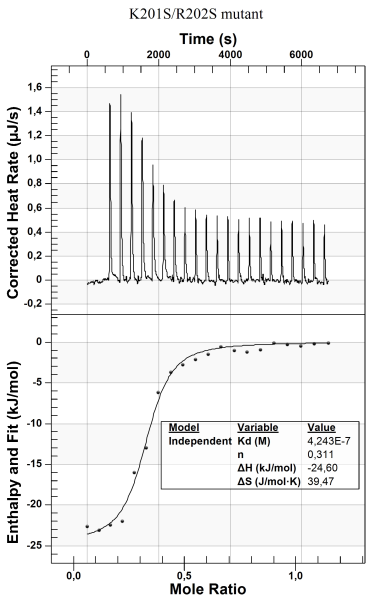

[K201S/R202S]ExtHD

Supplementary Figure 5. Representative ITC thermograms for the titration of ExtHD, mutants, HD and *N*-ter octadecapeptide with heparin in 50 mM NaH_2PO_4, 100 mM NaCl, pH 7.4 at 25°C. Experiments were acquired with a Nano ITC calorimeter (TA Instruments, New Castle, DE, USA) and analyzed with the Nanoanalyze software provided by the supplier. The data shown have been reproduced (n=3).

Supplementary Table 1. Interaction thermodynamics of ExtHD proteins, mutated in the region 189-204 (RKPKKKNPNKEDKRPR), with 12 kDa heparin studied by isothermal titration calorimetry (ITC). The proteins were titrated with the polysaccharide at 25°C in 50 mM NaH_2PO_4 (pH 7.4), 100 mM NaCl. Results are shown as mean ± SD (n=2).

Protein	Kd (nM)	ΔH (kJ / mol)	-TΔS (kJ / mol)	n (prot/polysacch.)
ExtHD	120 ± 18	-41 ± 2	-1.0 ± 1.0	2.9 ± 0.6
K193S/K194S	1010 ± 200	-34 ± 1	0.7 ± 0.1	3.0 ± 0.5
K201S/R202S	925 ± 175	-28 ± 1	-6.0 ± 1.0	3.3 ± 0.3
R189S/K190S	628 ± 6	-26 ± 1	-9.0 ± 1.0	2.7 ± 0.2

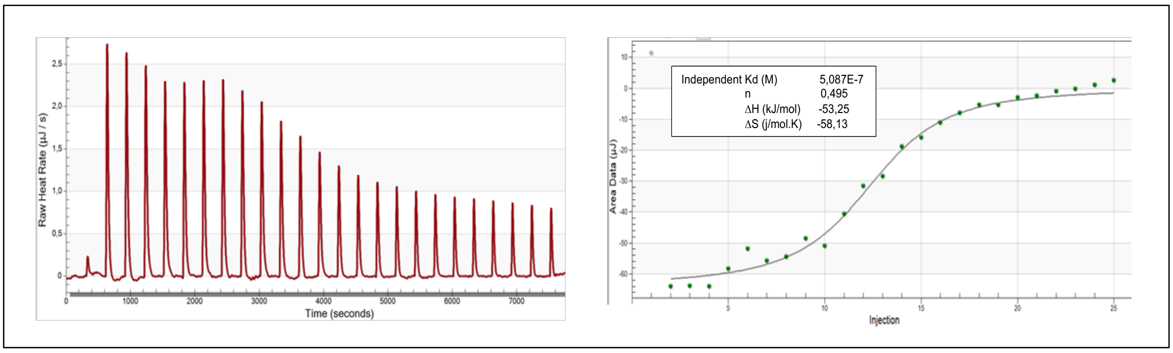

Supplementary Figure 6. Representative ITC thermogram for the titration of the full-length En2 protein with heparin in 50 mM NaH_2PO_4, 100 mM NaCl, pH 7.4 at 25°C. Experiments were acquired with a Nano ITC calorimeter (TA Instruments, New Castle, DE, USA) and analyzed with the Nanoanalyze software provided by the supplier. The data shown have been reproduced (n=3).

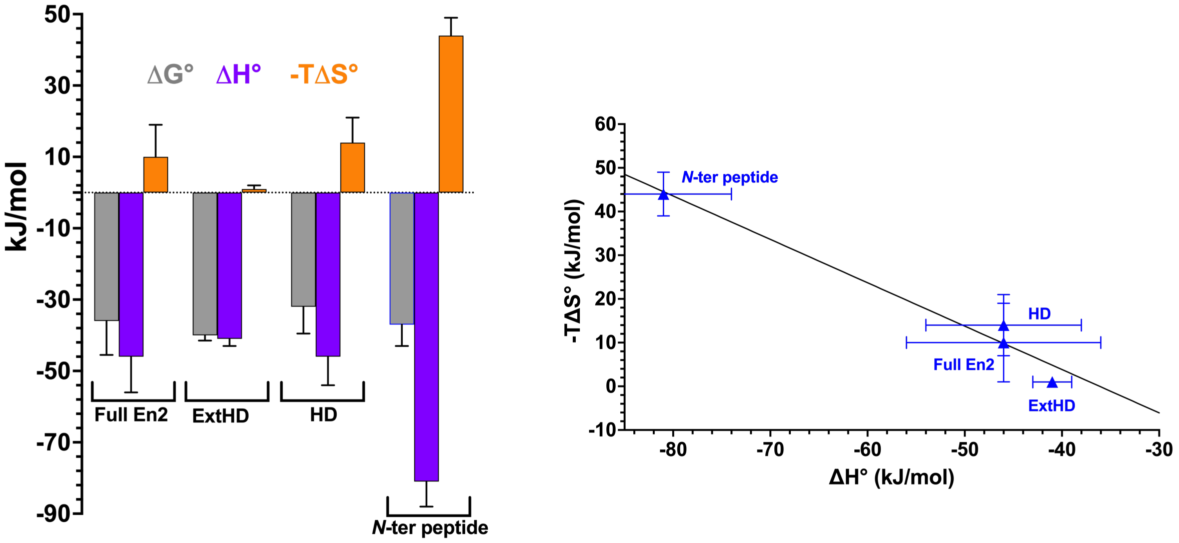

Supplementary Figure 7. Thermodynamic parameters of heparin (HI) interaction with En2 fragments obtained from ITC experiments. (Left) Thermodynamic profiles of full-length En2, ExtHD, HD, and *N*-ter peptide in complexes with HI, showing ΔG° (dark gray), ΔH° (blue), and −TΔS° (orange). (Right) Plot of −TΔS° versus ΔH° indicating possible enthalpy−entropy compensation. The full-length En2 protein interacts with HI with a free energy (ΔG°) slightly higher than that of ExtHD. However, the enthalpy value (ΔH°) is lower for the full-length protein (-51 kJ/mol) compared to ExtHD (-41 kJ/mol), suggesting that the full-length protein interacts stronger (additional non covalent interactions) with the anionic polymer. This difference of enthalpy is however compensated for the full-length protein by a higher unfavorable entropy than ExtHD, which results in the apparent lower affinity of the full-length protein for HI. Since En2 contains a large intrinsically disordered *N*-terminal region that is shortened in the ExtHD analogue, the higher entropy penalty measured for the interaction of full-length En2 with HI may be explained by a higher loss in the conformational flexibility and degree of freedom of the GAG-binding region. This large disordered *N*-terminal region is not present in the ExtHD variant. More generally, the data shown in the right panel suggest that the binding of En2 protein analogues and peptide to heparin involves enthalpy-entropy compensation. The bars and dots are means ± SD (n=3 for each condition).

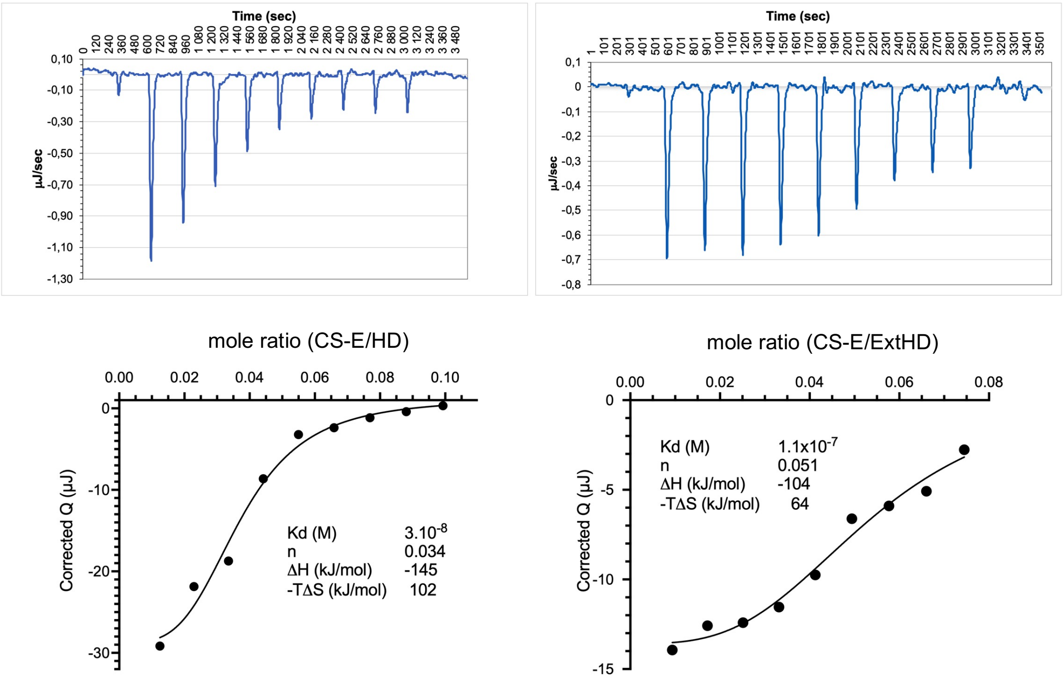

Supplementary Figure 8. Representative ITC thermograms for the titration of HD and ExtHD with CS-E in 50 mM NaH_2PO_4, 100 mM NaCl, pH 7.4 at 25°C. Experiments were acquired with a Nano ITC calorimeter (TA Instruments, New Castle, DE, USA) and analyzed with the Nanoanalyze software provided by the supplier. The data shown have been reproduced (n=3).

Analysis of the thermodynamic parameters of the interactions of En2 proteins with HI and CS-E GAG type by ITC. Because the size of CS-E differs from that of heparin, i.e. 12 kDa (~20 disaccharides) for heparin and 72 kDa (~135 disaccharides) for CS-E, we could only compare the energetics of binding for one particular polysaccharide chain, but cannot evaluate accurately the relative affinity of each protein for the two polysaccharides. The polyelectrolyte character of the polysaccharides (their length) is indeed a crucial parameter that influences the binding energetics of the protein. The increase of binding affinity with the length and charge density of these polyelectrolytes is expected owing to increased electrostatic interactions and Coulombic end effects[1] as previously reported for the formation of FGF-2-heparin[2] or Tat-heparin[3] complexes. Hence, to get access to the binding energy of only one protein molecule to one polysaccharide chain, the global free energy of binding was divided by the number of protein molecules bound per polysaccharide (stoichiometry determined in ITC experiments), but with the assumption or approximation that there is no cooperativity between proteins during binding to the polyelectrolyte and that all binding sites are similar.

Investigation of the GAG-binding properties of En2 proteins by NMR

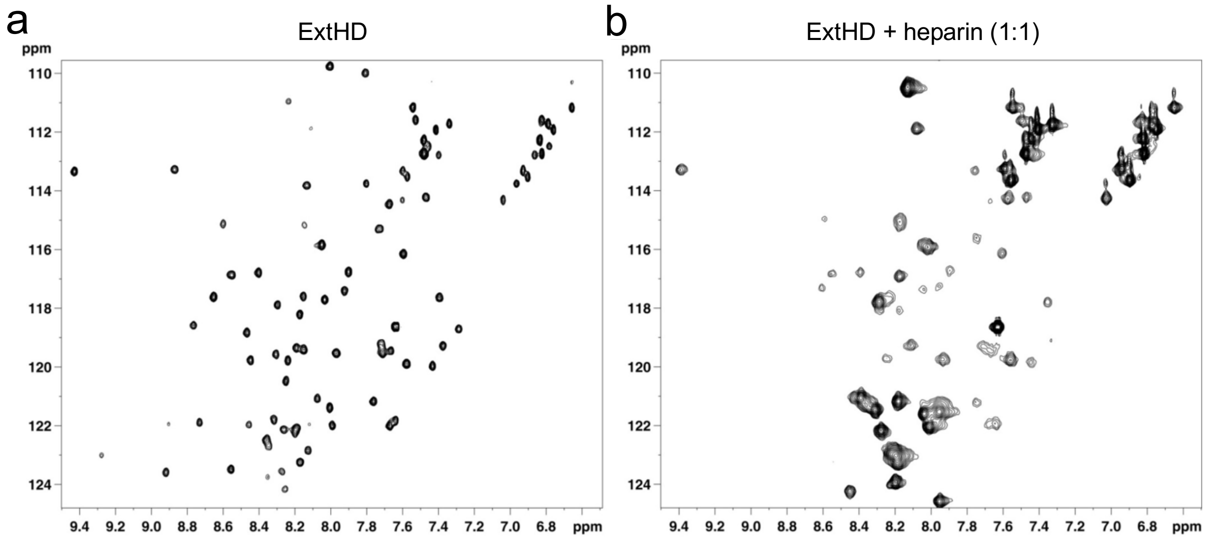

Supplementary Figure 9. ^1H-^{15}N HSQC of 150 µM ^{15}N-ExtHD in the absence (**a**) and presence (**b**) of one molar equivalent of unlabeled heparin.

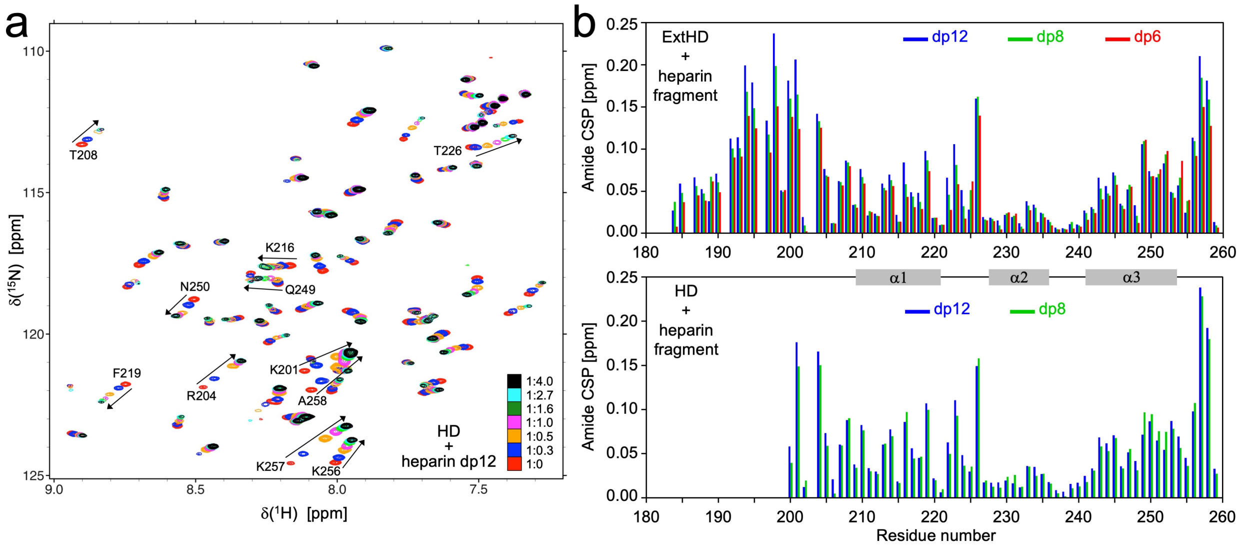

Supplementary Figure 10. Interaction of ExtHD and HD with heparin fragments dp12, dp8, and dp6 probed by NMR. **a**) Overlay of ^{1}H-^{15}N HSQC spectra obtained for ^{15}N-HD in the absence and presence of increasing amounts of heparin dp12. Residues exhibiting the highest perturbations are indicated in the spectra. Stoechiometry between ExtHD and heparin dp12 is : 1:0 (red), 1:0.3 (dark blue), 1:0.5 (orange), 1:1 (pink), 1:1.6 (green), 1:2.7 (light blue) and 1:4.0 (black). **b**) Average amide CSP of ExtHD and HD induced by the addition of saturating amounts of heparin dp12 (blue), dp8 (green), or dp6 (red).

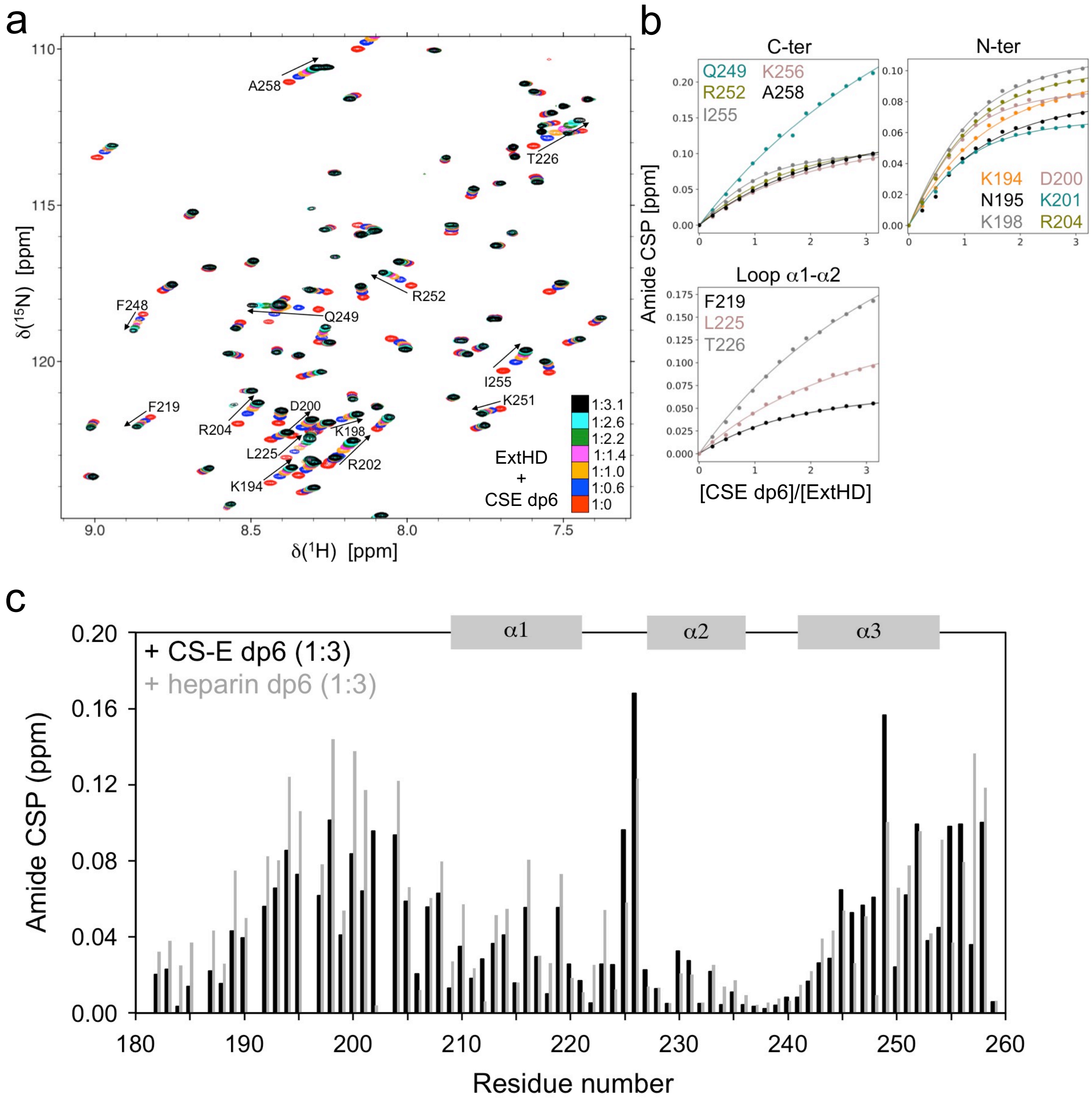

Supplementary Figure 11. Interaction of ExtHD with structurally defined CS-E dp6 probed by NMR. **a**) Overlay of ^{1}H-^{15}N HSQC spectra obtained for ^{15}N-ExtHD in the absence and presence of increasing amounts of CSE dp6. Residues exhibiting the highest perturbations are indicated in the spectra. Stoechiometry between ExtHD and CSE dp6 is : 1:0 (red), 1:0.6 (dark blue), 1:1 (orange), 1:1.4 (pink), 1:2.2 (green), 1:2.6 (light blue) and 1:3.1 (black). **b**) Saturation curves corresponding to the binding to CSE dp6 are shown for residues displaying the highest perturbations, with experimental data represented by points and non-linear curve fitting by lines. **c**) Amide chemical shift perturbations (CSP) of ExtHD induced by the presence of saturating amounts of CS-E dp6. For comparison, CSP values obtained with heparin dp6 are shown in grey.

Supplementary Table 2. Apparent K_d (K_d^{app}) at the residue level obtained from the non-linear curve fitting of NMR CSPs as a function of oligosaccharide/protein molar ratio. Values are given for representative residues of ExtHD in micromolar.

Residue	heparin dp12	heparin dp8	heparin dp6	CS-E dp6
**R189**		23 ± 5	99 ± 11	–
**K192**	5 ± 2	16 ± 3	52 ± 8	106 ± 17
**K193**	9 ± 3	24 ± 4	74 ± 8	113 ±15
**K194**	9 ± 3	22 ± 4	72 ± 7	113 ± 16
**N195**	5 ± 3	15 ± 3	46 ± 7	99 ± 21
**N197**	2 ± 1	5 ± 1	–	29 ± 15
**K198**	4 ± 2	5 ± 1	31 ± 4	67 ± 8
**D200**	2 ± 1	3 ± 1	14 ± 3	37 ± 5
**K201**	6 ± 2	10 ± 2	35 ± 6	49 ± 6
**R204**	1 ± 1	5 ± 1	23 ± 3	65 ± 8

Residue	heparin dp12	heparin dp8	heparin dp6	CSE dp6
**K216**	–	3 ± 1	18 ± 3	61 ± 7
**F219**	106 ± 21	180 ± 37	183 ± 16	184 ± 5
**R223**	> 1000	> 1000	> 1000	> 1000
**T226**	> 1000	> 1000	> 1000	782 ± 130
**Q249**	> 1000	> 1000	> 1000	> 1000
**R252**	61 ± 9	80 ± 17	96 ± 8	172 ± 17
**K256**	132 ± 30	444 ± 50	274 ± 14	263 ± 27
**K257**	67 ± 10	99 ± 7	133 ± 7	384 ± 42
**A258**	92 ± 19	174 ± 18	208 ± 7	309 ± 27

Model for the role of En2-GAG interaction in retinal axon guidance

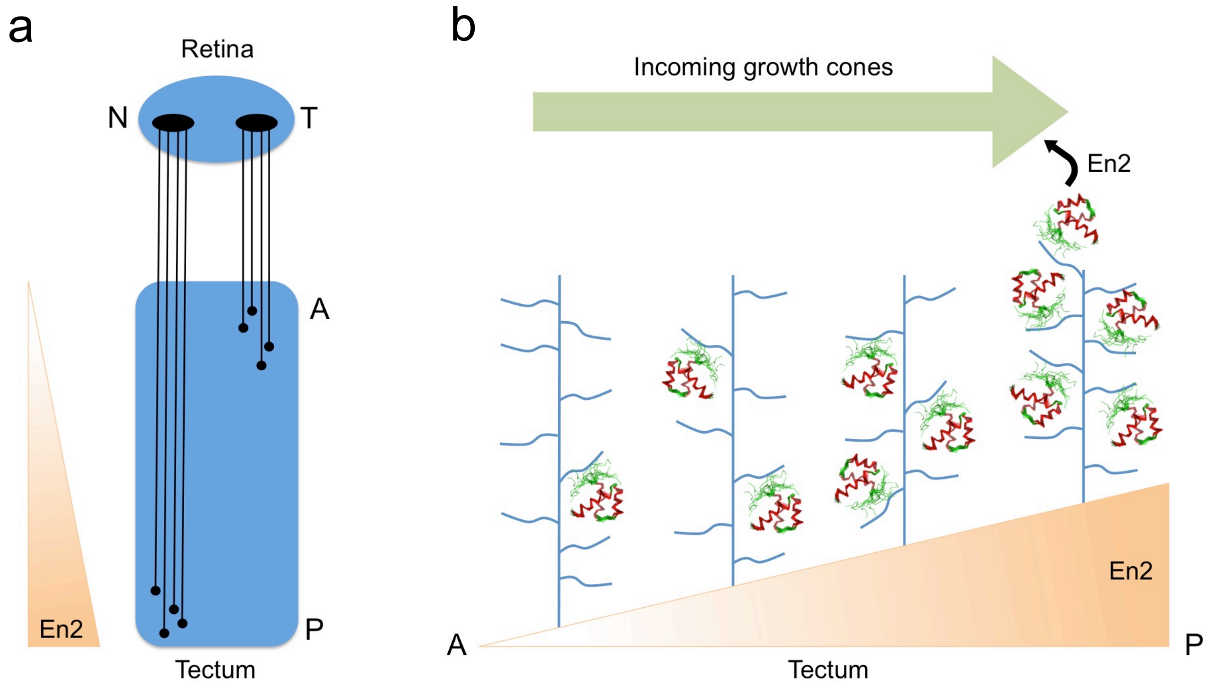

Supplementary Figure 12. En2 signaling in the retinotectal system. **a**) Schematic representation of the retinotectal system showing the projection of nasal (N) and temporal (T) axons onto the anteroposterior axis of the optic tectum (A, Anterior; P, posterior). Within the tectum, En2 and Ephrin A5 morphogens show an anterior-to posterior rising gradient that is required for proper positioning of nasal and temporal axons. **b**) Role of En2 graded expression in retinal axon guidance. En2 secreted from the tectum is maintained in the extracellular matrix at a concentration reflecting the secretion gradient, likely through electrostatic interactions with sulfated GAGs at the cell-surface that ensure a local confinement of the protein. Once internalized by growth cones in the posterior tectum, En2 stimulates the synthesis of several translational regulators, which results in Ephrin-mediated collapse of temporal growth cones.

Quantification of protein internalization by Mass Spectrometry

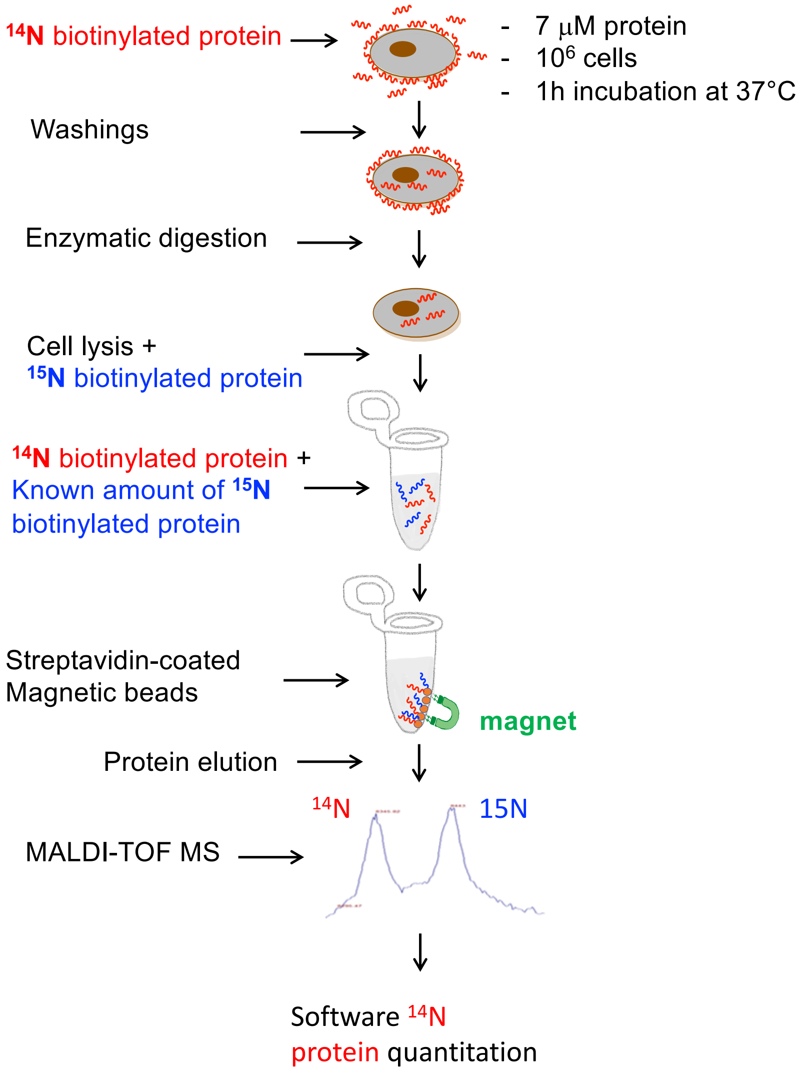

Supplementary Figure 13. Protocol used to quantify protein internalization in cells. See main text for details.

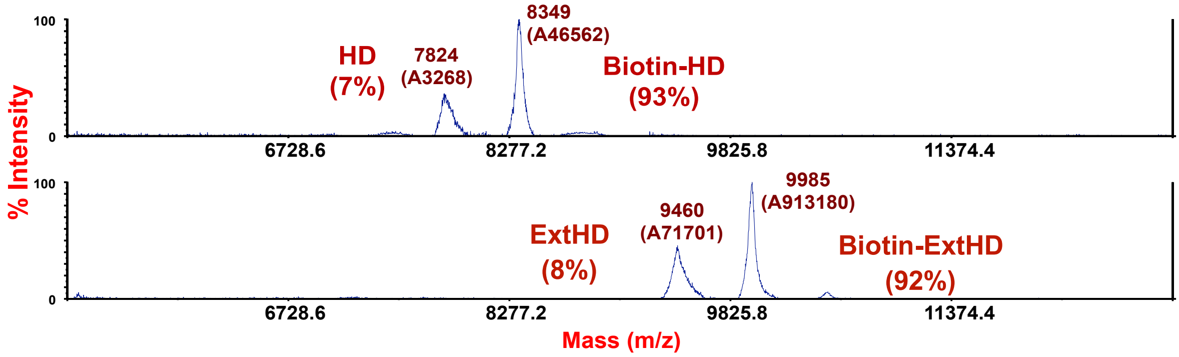

Supplementary Figure 14. Mass spectra (MH+) of ^{14}N biotin-labeled HD and ExtHD proteins. MS spectra were acquired on a Voyager DE-Pro MALDI-TOF apparatus in linear positive mode. The expected masses for biotin-HD and biotin-ExtHD are 8349 and 9985 Da, respectively.

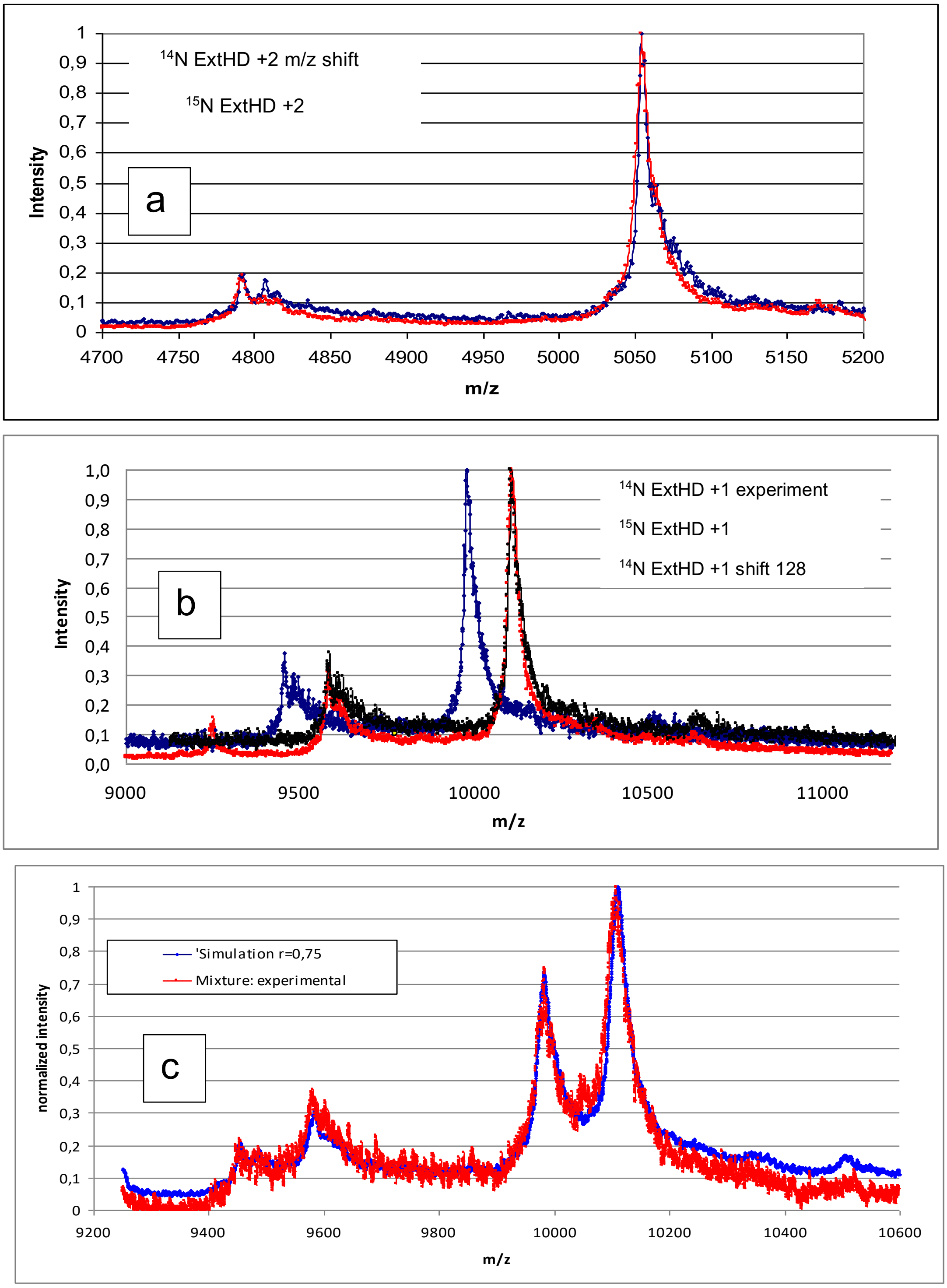

Supplementary Figure 15. **a)** and **b)** panels show the overlapped partial mass spectra of individual ^{14}N ExtHD and ^{15}N ExtHD samples. The two proteins are separated by $\Delta m = 128$ (mean labeling of the protein). On **a)**, the doubly charged ion of ^{15}N ExtHD, and that of ^{14}N ExtHD after m/z shift of 128/2. On **b)**, the singly charge of ^{15}N ExtHD (blue), and ^{14}N ExtHD (red) as well as that of the ^{14}N ExtHD (black) after m/z shift of 128. On **c)**, we show the fit of the ratio r = [^{14}N]/[^{15}N] for ExtHD protein using the peak of ^{14}N and ^{15} N separately recorded in the same conditions and mixed to form a ratio r and then compared to the experimental data of the mixture (red). The best adjustment for the MH$^+$ peak is obtained for a ratio r = 0.75 (blue). It should be noted that satellites peaks of the protein of interest can also be simulated and indicate a very close ratio r.

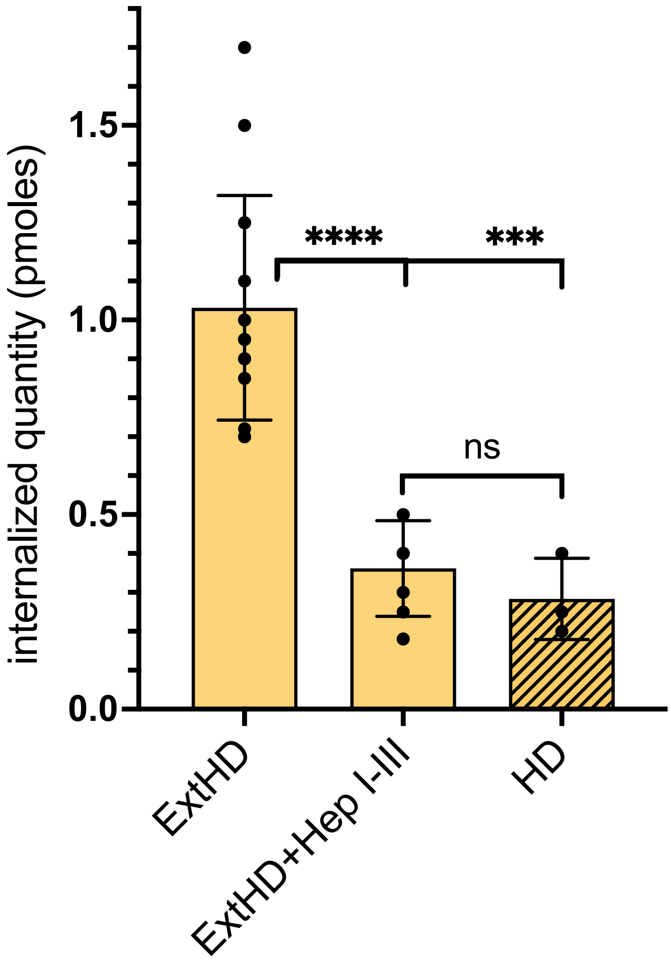

Supplementary Figure 16. Internalization quantity, 37°C, of ExtHD and HD in 10^6 SH-SY neuronal cells. Cell-surface HS dependency of ExtHD internalization is shown with heparinases I-III (Hep I-III) pre-treatment of SH-SY cells. Data are presented as mean ± SEM (n=15, 7 and 3, respectively ExtHD, ExtHD + HepI-III and HD). Comparison of the mean differences was performed with the unpaired t test (**** P<0.0001, ***P =0.0005).

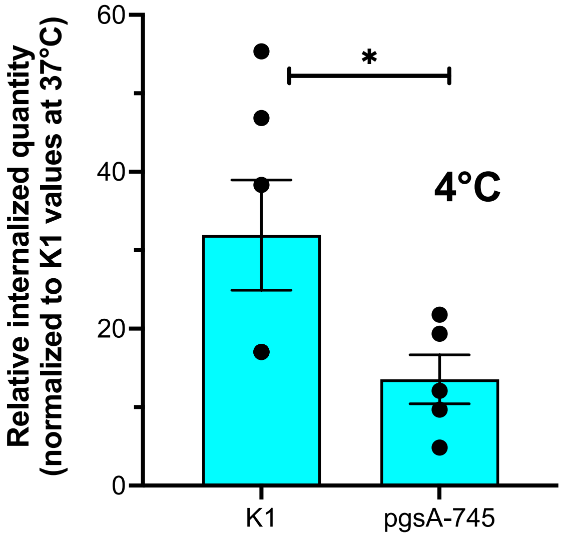

Supplementary Figure 17. Relative internalization of ExtHD at 4°C in K1 (n=6) and pgsA-745 (n=5) cells, normalized to internalization in CHO-K1 cells at 37°C, run in parallel. Data are presented as mean ± SEM. Comparison of the mean differences was performed with the unpaired t test (P = 0.0487, *significant)

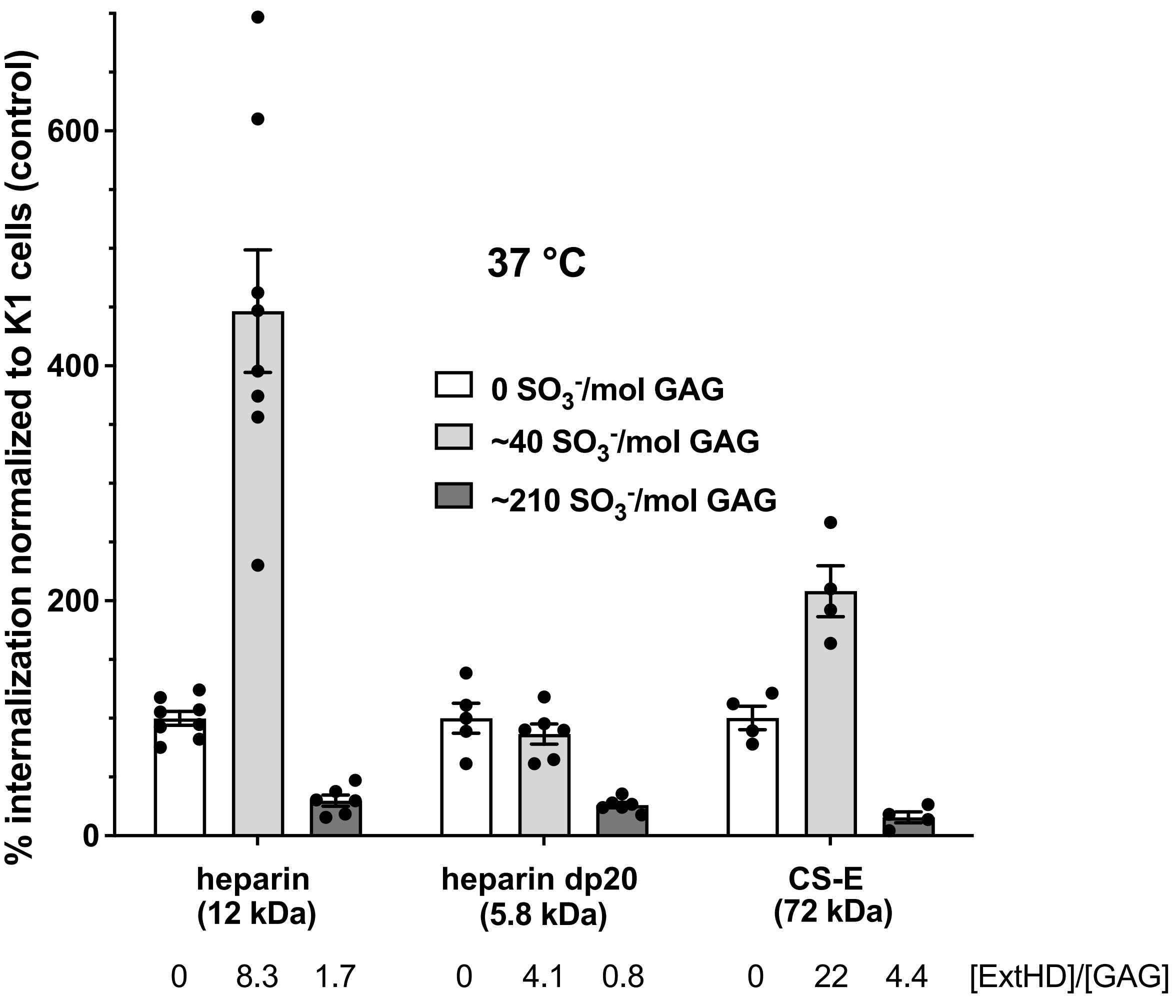

Supplementary Figure 18. Relative internalization of ExtHD at 37°C in CHO-K1 cells in the presence and absence of increasing amounts of full-length heparin, heparin dp20 or CS-E. Data were normalized to the intracellular quantity of ExtHD in control CHO-K1 cells at 37°C. The GAG molar concentration was adjusted to have similar amounts of sulfate groups in each experiment, taken that full-length heparin (12 kDa, 20 disaccharide units) has approximately 2.7 sulfate groups per disaccharide units[4] (~50 SO_3^-/mol), heparin dp20 (5.75 kDa, 10 disaccharide units) has half sulfate groups of heparin (~25 SO_3^-/mol), and that 60% of the disaccharide units from CS-E (72 kDa, ~120 disaccharide units) contains two sulfate groups (~150 SO_3^-/mol, information from Iduron, Cheshire, UK). The ExtHD/GAG molar ratio is indicated for each oligosaccharide concentration. Graphs shown mean ± SEM and every dot corresponds to one experiment (n=4-8).

Supplementary Table 3. Statistical analysis for significance between means of data presented in Fig. 2 (Quantity of internalized ExtHD or HD incubated at 7 μM with cells for 1 hr at 37°C). Data were analyzed by One-way ANOVA to compare the means of the different cell treatment conditions, followed by an unpaired t test to compare pairs of columns.

Parameter			
Table Analyzed	Cells + enzymatic treatments		
One-way analysis of variance			
P value	< 0.0001		
P value summary	***		
Are means signif. different? (P < 0.05)	Yes		
Number of groups	6		
F	94		
R squared	0,92		
Bartlett's test for equal variances			
Bartlett's statistic (corrected)	70		
P value	< 0.0001		
P value summary	***		
Do the variances differ signif. (P < 0.05)	Yes		
ANOVA Table	SS	df	MS
Treatment (between columns)	99000	9	11000
Residual (within columns)	8200	70	120
Total	110000	79	

Unpaired t test	Mean Diff.	t	Signifi-cant	Summary	95% confidence interval
K1 (control) vs K1 + ChABC	20	9.3	Yes	**** P<0.0001	-24.5 to -15.5
K1 (control) vs K1 + Hep II	48	21	Yes	**** P<0.0001	-52.9 to -43.1
K1 (control) vs K1 + ChABC, Hep II	58	32	Yes	**** P<0.0001	-61.8 to -54.2
K1 (control) vs K1 + Hep I-III	83	29	Yes	**** P<0.0001	-89.1 to -76.9
K1 (control) vs pgsA-745	83	31	Yes	**** P<0.0001	-88.7 to -77.3
K1 + ChABC vs K1 + Hep II	28	15	Yes	**** P<0.0001	-32.1 to -23.8
K1 + ChABC vs K1 + ChABC, Hep II	38	25	Yes	**** P<0.0001	-41.2 to -34.8
K1 + ChABC vs K1 + Hep I-III	63	23	Yes	**** P<0.0001	-68.7 to -57.3
K1 + ChABC vs pgsA-745	63	27	Yes	**** P<0.0001	-68.0 to -58.0
K1 + Hep II vs K1 + ChABC, Hep II	10	7.8	Yes	**** P<0.0001	-12.8 to -7.2
K1 + Hep II vs K1 + Hep I-III	35	11	Yes	**** P<0.0001	-41.6 to -28.4
K1 + Hep II vs pgsA-745	35	15	Yes	**** P<0.0001	-40.3 to -29.7
K1 + ChABC, Hep II vs K1 + Hep I-III	25	10	Yes	**** P<0.0001	-30.1 to -19.9
K1 + ChABC, Hep II vs pgsA-745	25	14	Yes	**** P<0.0001	-29.0 to - 21.0
K1 + Hep I-III vs pgsA-745	0.0	0.0	No	ns, P>0.9999	-7.2 to 7.2

Confocal imaging of En2 full-length protein internalization

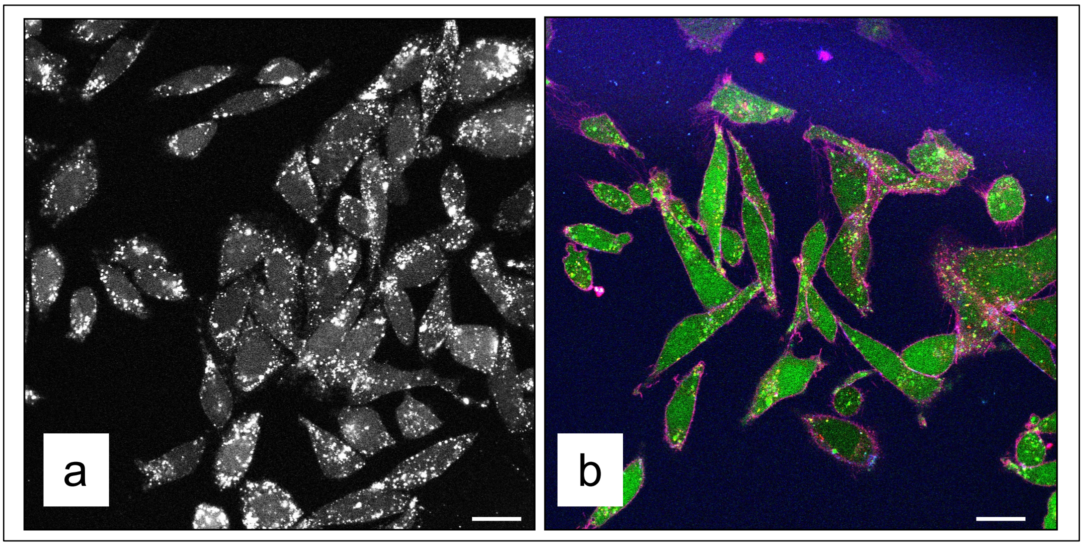

Supplementary Figure 19. Confocal imaging of fluorescent En2 internalization in CHO-K1 cells. Cells (2×10^4 per well) were plated on µ-slide six-well plates (Ibidi). After 24 h, medium was removed and cells were incubated with the fluorescent CF-En2 protein (1 µM) diluted in DMEM for 30 min at 37°C before visualization. Cells were analyzed either directly to visualize cell-surface staining or following addition of Trypan Blue (TB, 0.1% final concentration), an efficient quencher of all extracellular carboxyfluorescein (CF), fluorescence and a marker of permeabilized cells, to visualize only the intracellular staining in live cells. Images were acquired with CSUX1-A1 Yokogawa spinning disk coupled to a Zeiss Axio observer Z1Nikkon Ti Eclipse inverted microscope equipped with a Evolve EMCCD camera (Roper) and a 63× (1.45 Oil; WD:0.17 mm) objective. Two independent experiments are shown in the presence of TB: **a**) only FITC fluorescence (white) is shown; **b**) FITC (green) is shown together with TB fluorescence (pink, Ex. 640 nm, Em. 700 nm), which delineates the plasma membrane and attests to cell viability. Scale bar : 20 µm. Experiments have been performed 3 times with comparable results (a and b are two independent experiments).

References

1. Zhang W, Bond JP, Anderson CF, Lohman TM, Record MT (1996) Large electrostatic differences in the binding thermodynamics of a cationic peptide to oligomeric and polymeric DNA. Proc Natl Acad Sci USA 93(6):2511–2516.
2. Arakawa T, Wen J, Philo JS (1994) Stoichiometry of heparin binding to basic fibroblast growth factor. Arch Biochem Biophys 308(1):267–273.
3. Rusnati M, et al. (1999) Multiple interactions of HIV-I Tat protein with size-defined heparin oligosaccharides. J Biol Chem 274(40):28198–28205.
4. Toida T, et al. (1997) Structural differences and the presence of unsubstituted amino groups in heparan sulphates from different tissues and species. Biochem J 322(Pt2):499-506.
