# Supplementary material for: A cationic motif upstream Engrailed2 homeodomain controls cell internalization through selective interaction with heparan sulfates
Source: Nat Commun. 2023 Apr 10;14:1998. doi: 10.1038/s41467-023-37757-6 (PMC10083169; doi:10.1038/s41467-023-37757-6)
Supplement: Supplementary file 3 — Description of additional supplementary files [file 41467_2023_37757_MOESM3_ESM.pdf]

Description of additional supplementary files

Supplementary Software file : MS Software developed for the deconvolution and analysis of the mass spectra for quantitation of protein internalization in cells.
