# Supplementary material for: A cationic motif upstream Engrailed2 homeodomain controls cell internalization through selective interaction with heparan sulfates
Source: Nat Commun. 2023 Apr 10;14:1998. doi: 10.1038/s41467-023-37757-6 (PMC10083169; doi:10.1038/s41467-023-37757-6)
Supplement: Supplementary file 4 — Supplementary Software file [file 41467_2023_37757_MOESM4_ESM.zip › isopro3/readme.rtf]

IsoPro 3.0


·	Improved look with enhanced performance (>10x faster)
·	Improved formula entry options
	Direct Entry of Molecular Formula
	Entry of Amino Acid Content
	Import of PIR format files
·	Improved support for enriched/depleted isotopes
·	Improved control of simulation parameters
·	Improved Spectrum Display
	Completely User Customizable
	Print Preview
	Clipboard Support
·	Improved Peak List with printing and clipboard support


This is the latest release of IsoPro, and is a dramatic shift from v. 2.1. The biggest change is getting away from Visual Basic, and using Borland's Delphi for development. Visual Basic was interpretted and slow, and VB4.0 is worse. Delphi includes a true compiler, which means without algorithm improvements, an automatic gain of 10x in speed is realized, and fewer DLL's are needed to get things going.  Compared to v 2.1, a lot of less-used features are gone, but a lot of new improvements have been added.

A New Look:
This version uses the Borland "Notebook" motif, instead of the multi-form approach of v 2.0. This allows quicker access to the features you want to access. More of the simulation parameters are also now user adjustable. This is important when attempting to produce accurate results. The amino-acid input page is also now available. This makes simulating peptides and proteins much simpler when you only have the sequence.

A New Algorithm:
OK, it's still the Yergey algorithm, but after using it for a couple of years, I think I've finally figured out how it works. The v 2.0 implementation had a bug which would cause very premature pruning of the peak list, especially for metallic simulations. This was somewhat fixed in v 2.1, and is even better here. Because of the modified pruning strategy, this version won't give the exact same results as the last version, but the new results should be more accurate. This version also uses a recursive call to permut the isotopes, instead of nested loops. This means that you are no longer limited to an element with 10 isotopes or less (yeh, no one will ever use this feature).  Also, this version uses a linked-list instead of arrays, so much less memory is wasted.

A New Spectrum:
IsoPro now uses a professional plotting tool, which provides complete customization, zooming, clipboard support and print preview.  If you still don't like the way the data is plotted, you can now copy the spectrum data points to the clipboard in ASCII format so you can plot it in the program of your choice.

Installation:
	No setup.exe here.  Isopro needs only one file besides the executable, and that is the default periodic table information (table.txt).  Make sure these two files are in the same directory and you are ready go.

Registration:
	All MS/MS Software is available for a free 30 day evalutation.  You are free to redistribute the unregistered version of the program, but you must use the original zip archive which includes this file.  If you find the software useful, and choose to continue using it, you are obligated to pay the registration fee.

IsoPro 3.0:  $25/copy ($15 Academic)
FragPro 1.0: $15/copy ($10 Academic)

Registration entitles the user to one fully functional version of the software (downloadable at time of registration), including help files in HTML format, email support, as well as free upgrades to the current version (3.x for IsoPro and 1.x for FragPro).  There is an additional $5 fee if you wish to have the software mailed on a floppy disk.  Please make checks payable to Mike Senko.  Please include an email address for electronic delivery of the registered version or a postal address for delivery of a floppy.  Mail your checks to:

Mike Senko
243 Buena Vista Ave. #502
Sunnyvale, CA 94086

DISCLAIMER:  EVALUATOR OR PURCHASER ASSUMES ALL RESPONSIBILITIES AND RISKS ASSOCIATED WITH THE USE OF THESE SOFTWARE PRODUCTS. THE AUTHOR OF THIS SOFTWARE PRODUCT ASSUME NO RESPONSIBILITY OR LIABILITY FOR ANY ACTIONS ASSOCIATED WITH THIS SOFTWARE PRODUCT.

msmssoft@aol.com
